# Design, synthesis and theoretical simulations of novel spiroindane-based enamines as *p*-type semiconductors

**DOI:** 10.1098/rsos.232019

**Published:** 2024-05-08

**Authors:** Sarune Daskeviciute-Geguziene, Maryte Daskeviciene, Kristina Kantminienė, Vygintas Jankauskas, Egidijus Kamarauskas, Alytis Gruodis, Smagul Karazhanov, Vytautas Getautis

**Affiliations:** ^1^ Department of Organic Chemistry, Kaunas University of Technology, Kaunas 50254, Lithuania; ^2^ Department of Physical and Inorganic Chemistry, Kaunas University of Technology, Kaunas, 50254, Lithuania; ^3^ Institute of Chemical Physics, Vilnius University, Vilnius, 10257, Lithuania; ^4^ Institute for Energy Technology (IFE), Kjeller 2027, Norway

**Keywords:** spirobisindane, enamines, hole-transporting materials, solar cells

## Abstract

The search for novel classes of hole-transporting materials (HTMs) is a very important task in advancing the commercialization of various photovoltaic devices. Meeting specific requirements, such as charge-carrier mobility, appropriate energy levels and thermal stability, is essential for determining the suitability of an HTM for a given application. In this work, two spirobisindane-based compounds, bearing terminating hole transporting enamine units, were strategically designed and synthesized using commercially available starting materials. The target compounds exhibit adequate thermal stability; they are amorphous and their glass-transition temperatures (>150°C) are high, which minimizes the probability of direct layer crystallization. V1476 stands out with the highest zero-field hole-drift mobility, approaching 1 × 10^−5^ cm^2^ V s^−1^. To assess the compatibility of the highest occupied molecular orbital energy levels of the spirobisindane-based HTMs in solar cells, the solid-state ionization potential (*I*_p_) was measured by the electron photoemission in air of the thin-film method. The favourable morphological properties, energy levels and hole mobility in combination with a simple synthesis make V1476 and related compounds promising materials for HTM applications in antimony-based solar cells and triple-cation-based perovskite solar cells.

## 1. Introduction

Since the use of the first device incorporating an organic semiconductor (OS) in the latter part of the twentieth century [1], a number of innovative devices using organic materials for charge transport have been developed. These include organic field-effect transistors (OFETs) [2], organic light-emitting diodes (OLEDs) [3] and various types of organic or hybrid solar cells (SCs) [4–6]. Typically, these devices comprise multiple layers of OSs, each serving a distinct function, such as light emission, light absorption and charge transfer [6,7].

In recent decades, antimony- and triple-cation-based SCs have been significantly improved, resulting in notably enhanced efficiencies. Hole-transporting materials (HTMs) play a pivotal role in all types of SCs as they transport photogenerated holes to contact [8,9]. High hole-drift mobility, appropriate energy levels and the capacity to create high-quality thin films are essential attributes of effective HTMs [10–12]. Small molecules as HTMs have attracted a lot of attention due to their well-defined structures, facile synthesis/purification, high chemical purity and reproducible film forming ability [13,14]. Until recently, the organic low-molecular-mass spiro-OMeTAD, derived from 9,9′-spirobifluorene, has been central to the development of highly efficient SCs. The following factors are characteristic of spiro-OMeTAD. (i) It has a large band gap (approx. 3.0 eV) and a relatively deep-lying HOMO energy level, which provides good electronic alignment with the perovskite layers; its band gap can be further tuned to the electronic structure of a chosen perovskite [15,16]. (ii) Spiro-OMeTAD benefits from a thoroughly researched synthesis and solution method, making it advantageous for manufacturing both rigid and flexible SCs on a large scale. (iii) Its high melting point contributes to the thermal stability of a device [17,18]. (iv) Pure spiro-OMeTAD hole transporting layer (HTL) exhibits low conductivity and hole mobility [19]. A commonly used method includes the use of additives, such as 4-*tert*-butylpyridine (TBP) and LiTFSI, to enhance electrical properties of spiro-OMeTAD films [20–22]. For these reasons, HTMs based on spiro-OMeTAD undeniably have a significant impact on the advancement of antimony-based and triple-cation-based SCs.

Despite its numerous advantages, the crystallization tendency of spiro-OMeTAD, owing to the symmetry of its central spirobifluorene fragment [23], limits its ability to form films, potentially impacting device stability [24]. Taking into account this criterion, a logical approach involves removing two arms of the spirobifluorene core [25] to transform it into the spirobisindane core [26] with reduced symmetry. Additionally, the advantages of spirobisindane are its synthesis from a cheap commercially available bisphenol A in high yield and simple purification.

It is imperative to synthesize new OSs via simple and green chemistry methods without compromising the efficiency of the solar cell [27–29]. One of the approaches is the preparation of enamines by a facile condensation reaction because condensation chemistry offers a promising alternative to palladium-catalysed reactions since it produces water as the only by-product and eliminates the need for expensive catalysts. In addition, it includes facile product workup and purification [30–32]. Furthermore, enamines have been successfully applied in antimony- or triple-cation-based SCs with and without additives, showing excellent efficiency and long-term stability [32–34].

**Figure 1.**
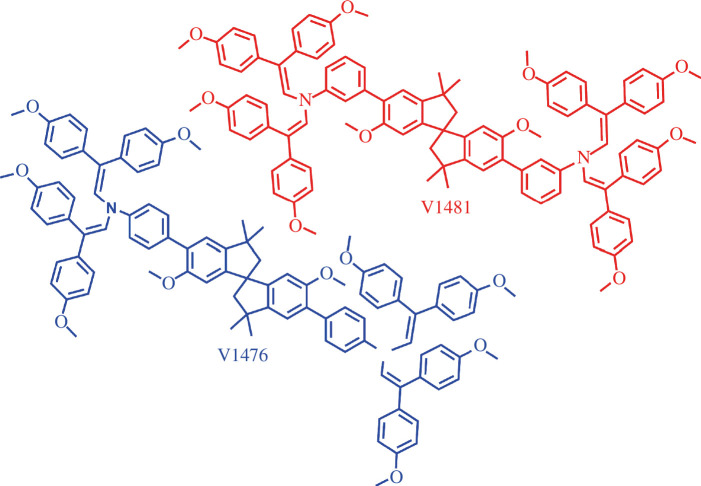
Chemical structures of the synthesized hole-transporting materials V1476 and V1481.

This study is devoted to further exploration of the enamine family HTMs using spirobisindane as the central core. By combining different aniline substituents, two spirobisindane-based HTMs shown in figure 1 were designed and synthesized from commercially available compounds without the use of costly metal catalysts. The optical, thermal and electrophysical properties of V1476 and V1481 were thoroughly investigated. Both HTMs exhibited high thermal stability and relatively high hole-drift mobility, making them viable candidates for application as HTMs in SCs. Density functional theory (DFT) Cam-B3LYP method and 6-31G(d) basis set (supplemented with polarization functions (d)) were used for ground-state optimization that supplemented the experimental study.

## 2. Experimental section

### 2.1. Chemical reagents and instruments

Information about chemical reagents used for synthesis of the target compounds V1476 and V1481 and instruments used for their characterization is provided in the electronic supplementary material.

### 2.2. Synthesis

#### 2.2.1. 3,3,3*ʹ*,3′-Tetramethyl-1,1′-spirobisindane-6,6′-diol (**1**), 6,6′-dimethoxy-3,3,3′,3′-tetramethyl-1,1′-spirobisindane (**2**) and 5,5′-dibromo-6,6′-dimethoxy-3,3,3′,3′-tetramethyl-1,1′-spirobisindane (**3**)

3,3,3′,3′-Tetramethyl-1,1′-spirobisindane-6,6′-diol (**1**), 6,6′-dimethoxy-3,3,3′,3′-tetramethyl-1,1′-spirobisindane (**2**) and 5,5ʹ-dibromo-6,6′-dimethoxy-3,3,3′,3′-tetramethyl-1,1′-spirobisindane (**3**) were prepared according to the synthesis procedures described in [35]. Detailed synthesis procedures are provided in the electronic supplementary material.

#### 2.2.2. 5,5′-Bis(4-aminophenyl)-6,6′-dimethoxy-3,3,3′,3′-tetramethyl-1,1′-spirobisindane (**4**)

A mixture of **3** (1 g, 2 mmol, 1 eq) and 4-(4,4,5,5-tetramethyl-1,3,2-dioxaborolan-2-yl)aniline (1 g, 4.5 mmol, 2.2 eq) in 40 ml of anhydrous solvent mixture of tetrahydrofuran (THF) and toluene (1:1) was purged with argon for 10 min. Afterwards, sodium hydroxide (1.2 g, 30.3 mmol, 15 eq) and PdCl_2_(PPh_3_)_2_ (0.7 g, 1 mmol, 0.5 eq) were added, and the reaction mixture was heated under reflux under argon atmosphere for 23 h. After the reaction mixture was cooled to room temperature, it was filtered, and solvent was evaporated *in vacuo*. The crude product was purified by column chromatography (THF/*n*-hexane; 8:17 v/v) to obtain **4** as a pale yellow solid. Yield 0.94 g (89.5%). ^1^H NMR (400 MHz, DMSO-*d*_6_) δ 7.18 (*d*, *J* = 8.2 Hz, 4H), 7.05 (*s*, 2H), 6.59 (*d*, *J* = 8.2 Hz, 4H), 6.39 (*s*, 2H), 5.03 (*s*, 4H), 3.58 (*s*, 6H), 2.32 (*d*, *J* = 12.8 Hz, 2H), 2.22 (*d*, *J* = 12.8 Hz, 2H), 1.39 (*s*, 6H), 1.32 (*s*, 6H). ^13^C NMR (101 MHz, DMSO) δ 156.46, 149.21, 147.92, 144.56, 130.39, 130.32, 126.46, 123.54, 113.87, 107.12, 59.66, 58.08, 56.21, 42.99, 31.94, 30.90.

#### 2.2.3. 5,5ʹ-Bis/{4[(4-methoxyphenyl)etenyl]amino}phenyl/-6,6′-dimethoxy-3,3,3′,3′-tetramethyl-1,1′-spirobisindane (**V1476**)

To a solution of **4** (0.6 g, 1.2 mmol, 1 eq) in THF (5 ml + volume of the Dean–Stark trap), (+/−)camphor-10-sulfonic acid (0.27 g, 1.2 mmol, 1 eq) was added, and the reaction mixture was heated under reflux for 20 min. Afterwards, 2,2-bis(4-methoxyphenyl)acetaldehyde (1.8 g, 6.9 mmol, 6 eq) was added, and heating under reflux was continued with the removal of water using a Dean–Stark trap for 40 min. After cooling down, the reaction mixture was poured into 15-fold excess of ethanol. The obtained precipitate was filtered off and washed with water and ethanol. The crude product was purified by column chromatography (THF/*n*-hexane; 6.5:18.5 v/v) to obtain V1476 as a yellow solid. Yield 1.05 g (61.8%). ^1^H NMR (400 MHz, DMSO-*d*_6_) δ 7.41 (*d*, *J* = 8.0 Hz, 4H), 7.09 (*s*, 2H), 7.00 (*d*, *J* = 8.0 Hz, 4H), 6.94–6.83 (*m*, 16H), 6.64 (*d*, *J* = 8.6 Hz, 8H), 6.42 (*s*, 2H), 6.38 (*d*, *J* = 8.6 Hz, 8H), 5.71 (*s*, 4H), 3.81 (*s*, 12H), 3.68 (*s*, 12H), 3.57 (*s*, 6H), 2.31 (*d*, *J* = 12.6 Hz, 2H), 2.24 (*d*, *J* = 12.6 Hz, 2H), 1.37 (*s*, 6H), 1.32 (*s*, 6H). ^13^C NMR (101 MHz, DMSO) δ 159.24, 158.90, 156.49, 150.14, 144.67, 134.06, 132.38, 130.79, 130.60, 129.52, 128.80, 126.59, 116.22, 114.46, 113.55, 107.09, 59.53, 58.24, 56.18, 55.75, 55.49, 43.03, 31.93, 30.84. Anal. calcd for C_99_H_94_N_2_O_10_: C, 80.79; H, 6.44; N, 1.9; found: C, 80.55; H, 6.49; N, 1.9. C_99_H_94_N_2_O_10_ [M^+^] exact mass = 1470.69, MS (ESI) = 1472.10.

#### 2.2.4. 5,5ʹ-Bis(3-aminophenyl)-6,6′-dimethoxy-3,3,3′,3′-tetramethyl-1,1′-spirobisindane (**5**)

A mixture of **3** (0.7 g, 1.4 mmol, 1 eq) and 3-(4,4,5,5-tetramethyl-1,3,2-dioxaborolan-2-yl)aniline (0.7 g, 3.1 mmol, 2.2 eq) in 28 ml of anhydrous solvent mixture of THF and toluene (1:1) was purged with argon for 10 min. Afterwards, sodium hydroxide (0.9 g, 21.2 mmol, 15 eq) and PdCl_2_(PPh_3_)_2_ (0.5 g, 0.7 mmol, 0.5 eq) were added, and the solution was heated under reflux under argon atmosphere for 22 h. After cooling down, the reaction mixture was filtered and solvent was evaporated *in vacuo*. The crude product was purified by column chromatography (THF/*n*-hexane; 2:3 v/v) to obtain **5** as a pale yellow solid. Yield 0.68 g (93.2%). ^1^H NMR (400 MHz, DMSO-*d*_6_) δ 7.08 (*s*, 2H), 7.03 (*t*, *J* = 7.6 Hz, 2H), 6.69 (*s*, 2H), 6.62 (*d*, *J* = 7.4 Hz, 2H), 6.52 (*d*, *J* = 7.4 Hz, 2H), 6.44 (*s*, 2H), 5.03 (*s*, 4H), 3.59 (*s*, 6H), 2.34 (*d*, *J* = 13.0 Hz, 2H), 2.26 (*d*, *J* = 13.0 Hz, 2H), 1.41 (*s*, 6H), 1.34 (*s*, 6H). ^13^C NMR (101 MHz, DMSO) δ 156.55, 150.14, 148.69, 144.47, 139.85, 130.75, 128.74, 124.09, 117.69, 115.55, 112.82, 107.15, 59.57, 58.19, 56.25, 43.02, 31.94, 30.87.

#### 2.2.5. 5,5ʹ-Bis/{3[(4-methoxyphenyl)etenyl]amino}phenyl/-6,6′-dimethoxy-3,3,3′,3′-tetramethyl-1,1′-spirobisindane (**V1481**)

To a solution of **5** (0.6 g, 1.2 mmol, 1 eq) in THF (5 ml + volume of the Dean–Stark trap), (+/−)camphor-10-sulfonic acid (0.27 g, 1.2 mmol, 1 eq) was added, and the reaction mixture was heated under reflux for 20 min. Afterwards, 2,2-bis(4-methoxyphenyl)acetaldehyde (1.8 g, 6.9 mmol, 6 eq) was added, and heating under reflux was continued with the removal of water using a Dean–Stark trap for 40 min. After cooling down, the reaction mixture was poured into 15-fold excess of ethanol. The formed precipitate was filtered off and washed with water and ethanol. The crude product was purified by column chromatography (THF/*n*-hexane; 6.5:18.5 v/v) to obtain V1481 as a yellow solid. Yield 1.08 g (63.3%). ^1^H NMR (400 MHz, DMSO-*d*_6_) δ 7.32 (*t*, *J* = 7.8 Hz, 2H), 7.16–7.03 (*m*, 6H), 6.97–6.81 (*m*, 18H), 6.63 (*d*, *J* = 8.4 Hz, 8H), 6.46–6.30 (*m*, 10H), 5.73 (*s*, 4H), 3.78 (*s*, 12H), 3.67 (*s*, 12H), 3.52 (*s*, 6H), 2.29 (*d*, *J* = 12.8 Hz, 2H), 2.17 (*d*, *J* = 12.8 Hz, 2H), 1.34 (*s*, 6H), 1.27 (*s*, 6H). ^13^C NMR (101 MHz, DMSO) δ 159.20, 158.85, 156.42, 150.63, 145.68, 144.68, 140.27, 134.08, 132.39, 130.68, 130.59, 129.77, 128.75, 126.77, 118.07, 114.46, 113.54, 107.34, 59.46, 58.29, 56.23, 55.72, 55.47, 43.01, 31.88, 30.80. Anal. calcd for C_99_H_94_N_2_O_10_: C, 80.79; H, 6.44; N, 1.9; found: C, 80.59; H, 6.48; N, 1.9. C_99_H_94_N_2_O_10_ [M^+^] exact mass = 1470.69, MS (ESI) = 1472.18.

## 3. Results and discussion

### 3.1. Synthesis

**Figure 2.**
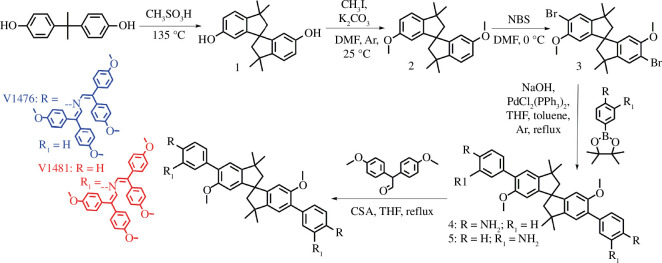
Synthesis route towards the target hole-transporting materials V1476 and V1481.

The overall synthesis procedure for the preparation of new HTMs V1476 and V1481 is depicted in figure 2. A readily available low-cost bisphenol A is used as a starting compound. In the simple initial cyclization step, bisphenol A was heated in methanesulfonic acid. Next, spirobisindane (**1**) was alkylated using iodomethane and a base in dimethylformamide as a solvent at room temperature. The intermediate product **2** was then brominated using *N*-bromosuccinimide, eliminating the need for aggressive bromine. To obtain enamines, an amino group was introduced into the molecule through an aqueous/THF/toluene twofold Suzuki cross-coupling procedure to yield precursors **4** and **5** with different benzene substitutions at the *para* and *meta* positions. Subsequently, the aminated precursors were condensed with the commercially available reagent 2,2-bis(4-methoxyphenyl)acetaldehyde in the presence of camphor sulfonic acid to produce the target products V1476 and V1481. Water was the only by-product, which was removed from the reaction mixture using a Dean–Stark trap. The chemical structures of the synthesized compounds were confirmed based on the ^1^H NMR, mass spectrometry, and elemental analysis data (electronic supplementary material, figures S1 and S2). It should be noted that attempts to synthesize the target analogue with the amino group at the *ortho* position failed. Presumably, steric hindrance prevented the formation of such an enamine derivative.

### 3.2. Thermal and optical properties

**Figure 3.**
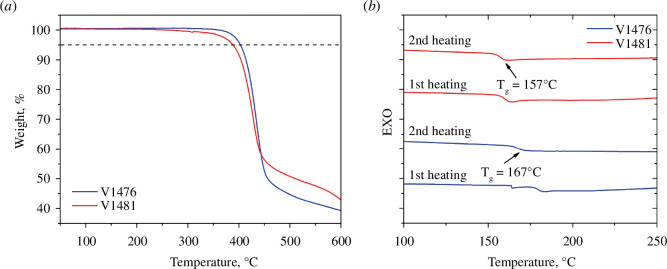
(*a*) Thermogravimetric analysis (TGA) data for V1476 and V1481 (heating rate of 10℃ min^−1^, N_2_ atmosphere). (*b*) First and second heating curves of differential scanning calorimetry (DSC) for V1476 and V1481 (scan rate 10°C min^−1^, N_2_ atmosphere).

The thermal characteristics of the HTMs were evaluated by thermogravimetric analysis (TGA) (figure 3*a*) and differential scanning calorimetry (DSC) (figure 3*b*) measurements. Understanding these characteristics is crucial, especially in the context of processing temperatures, as they can potentially impact the long-term stability of SCs. TGA has revealed that V1476 exhibits higher thermal stability with a decomposition temperature (*T*_dec_) of 403°C at 5% weight loss than *meta*-substituted HTM V1481 (*T*_dec_ = 389°C). Notably, both synthesized enamines possess higher *T*_dec_ than that of spiro-OMeTAD (*T*_dec_ = 288°C) [23]. The DSC measurements were employed to identify the thermal changes in the new HTMs. The results have demonstrated that the new compounds are entirely non-crystalline with only a glass transition temperature (*T*_g_) recorded (V1476 *T*_g_ = 167°C and V1481 *T*_g_ = 157°C). Interestingly, *T*_g_ of both synthesized HTMs are higher than that of spiro-OMeTAD (*T*_g_ = 124°C), indicating that the spirobisindane-based HTMs are likely to possess better morphological stability. Furthermore, it is worth noting that spiro-OMeTAD is not fully amorphous; it has a crystallization temperature and a melting point, factors that can compromise the long-term stability of SCs [23].

**Figure 4.**
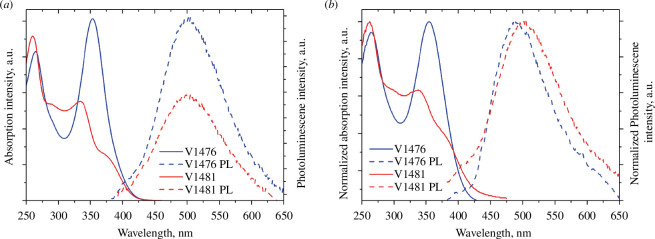
(*a*) UV−Vis absorption (solid line) and photoluminescence (dashed line) spectra of V1476 and V1481 in THF solutions (10^−4^ M). (*b*) UV−Vis absorption (solid line) and photoluminescence (dashed line) spectra of thin films of V1476 and V1481.

The ultraviolet–visible (UV–Vis) absorption spectra of spirobisindane-based HTMs were recorded in THF solutions and are depicted in figure 4*a*. Two major absorption peaks at approximately 265 and 360 nm are present in the spectra of both HTMs. The absorption peak at 265 nm corresponds to the localized π–π^*^ transitions originating from the central spirobisindane scaffold. The more intensive delocalization of the different conjugated substituents (*meta* and *para*) gives rise to longer wavelength peaks and indicates conjugated π–π^*^ and *n*–π^*^ transitions. The significant changes in molecular geometry of the synthesized molecules upon excitation have been proven by the presence of peaks at 500 nm, showing markedly large Stokes shifts of approximately 150 nm, in the photoluminescence spectra of both compounds. The optical gaps (*E*_g_) of new HTMs were calculated from the crossing of absorption and photoluminescence spectra of thin films (figure 4*b*) to be similar for both HTMs at approximately 3 eV (table 1). Notably, no shift in absorption can be observed in the spectra of the same compounds in solution in comparison with the ones of those acting as thin films. This one more advantageous property of the novel HTMs is likely attributed to their stereostructure. These compounds do not form aggregates in the layers, which is the usual form for use of such materials in SCs [36].

**Table 1.** Parameters of electronic excitations (transition energy ∆*E_n_* and corresponding oscillator strength *f_n_*) simulated using semiempirical TD method (for singlets).

compound	∆*E*_1_(S_0_→S_1_) (eV)	*f*_1_	∆*E*_2_(S_0_→S_2_) (eV)	*f*_2_	∆*E*_3_(S_0_→S_3_) (eV)	*f*_3_
V1476a	3.71	0.289	3.83	0.522	3.96	1.612
V1481a	3.84	0.266	3.85	0.904	4.14	0.428
V1481b	3.83	0.469	3.85	0.687	4.11	0.343

Furthermore, contact angle (*θ*) measurements were carried out to assess the hydrophobicity of the HTMs (electronic supplementary material, figure S3). No obvious difference in *θ* values between the films of the synthesized spirobisindane-based enamines V1476, V1481 and that of spiro-OMeTAD can be observed, implying that their surface hydrophobicity is almost the same. Therefore, it may be assumed that the device stability should be similar.

### 3.3. Theoretical calculations

**Figure 5.**
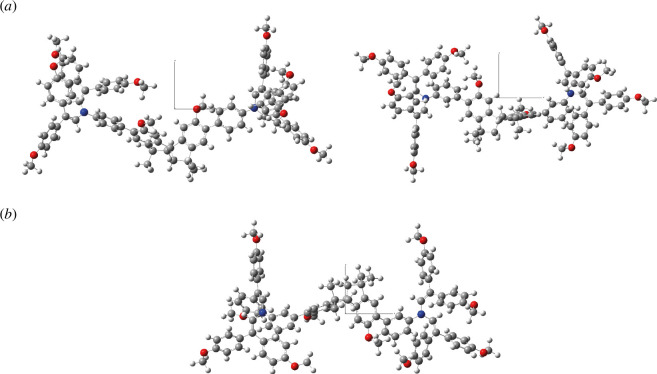
Most stable conformations of V1476 and V1481 obtained after ground-state energy optimization. B3LYP/6-31G(d). Projection *xy*.

Software *Gaussian 16* was used to determine the most probable molecular conformation using quantum chemistry methods. DFT Cam-B3LYP method and 6-31G(d) basis set (supplemented with polarization functions (d)) were used for ground-state optimization [37]. Due to the large volume of molecular structures, solvation effects were not considered in all cases. The three most probable molecular conformations are presented in figure 5. Total molecular symmetry is absent. Substituents are oriented in a chaotic manner resulting in a vast array of different conformers. All structures depicted in figure 5 were derived using the grad optimization technique, ensuring convergence of all parameters such as Maximum Force, RMS Force, Maximum Displacement and RMS Displacement.

Electronic excitations of fully optimized structures were simulated using the semiempirical TD method (for singlets). Table 1 presents the parameters of electronic excitations (transition energies ∆*E*_1_(S_0_→S_1_), ∆*E*_2_(S_0_→S_2_), ∆*E*_3_(S_0_→S_3_) and corresponding oscillator strengths *f*_1_*, f*_2_*, f*_3_) for all structures. The population of low-lying excited molecular states S_1_ and S_2_ was achieved through partially allowed transitions S_0_→S_*n*_*, n* = 1, 2 (oscillator strengths *f*_*n*_ > 0.2). The experimental absorption spectra of both solutions and thin films, as depicted in figure 4, exhibit excellent agreement with simulated spectra. Electronic supplementary material, figure S4, represents the molecular orbitals of V1476 and V1481 which are involved in ‘spectroscopic’ transitions (population of ‘spectroscopic’ states S_1_*,* S_2_). In all instances, the predominant and most significant electron jump of the CT transition (π–π*) type occurs between the highest occupied molecular orbital (HOMO) and the lowest unoccupied molecular orbital (LUMO). Electronic supplementary material, table S1, lists the spatial distributions of electron density for the HOMO-1, HOMO, LUMO and LUMO+1 of each compound, while transition parameters between molecular orbitals (MOs) related to the population of ‘spectroscopic’ state are detailed in table 1. Based on such simulations, it can be argued that the central core fragment (two pentarings oriented at an angle of about 80° instead of perpendicular) does not participate in CT excitations, and the molecular charge redistribution is provided between substituents only. In all cases, the orientation of the substituents (relative to each other) is not ideal, but the presence of many phenyl moieties associated with the single bond (each with no significant rotational barrier) creates the possibility of quite effective partially allowed charge redistribution.

### 3.4. Photoelectric properties

**Figure 6.**
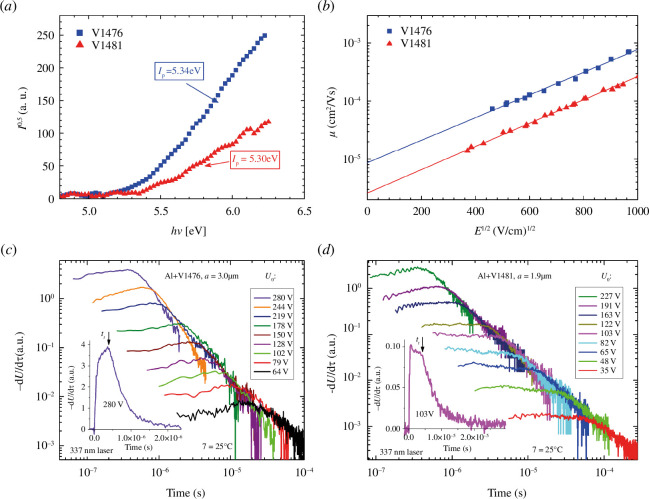
(*a*) Photoemission in air spectra of the charge transporting layers. (*b*) Electric field dependencies of the hole-drift mobility in V1476 and V1481. (*c*) Photocurrent XTOF transients of holes in V1476. (*d*) Photocurrent XTOF transients of holes in V1481.

The HOMO energy level of the material stands out as one of the most important parameters when selecting HTMs for device applications. To assess the compatibility of the HOMO energy levels of the spirobisindane-based HTMs for application in SCs, the solid-state ionization potential (*I*_p_) was measured through the electron photoemission in air of thin films (PESA) method. The experimental results are presented in figure 6*a*. *I*_p_ values for V1476 and V1481 are 5.34 and 5.3 eV, respectively. They are in the same range as the preferred *I*_p_ values (4.9–5.5 eV) of HTMs used in antimony-based and triple-cation-based perovskite SCs [34,38,39]. The LUMO energy level was determined by calculating *E*_ea_ (electron affinity, table 2) from the interaction of absorption and emission spectra of solid films after determination of the optical bandgap (*E*_g_).

**Table 2.** Thermal, optical and photophysical properties of V1476 and V1481.

Compound	*T*_g_ (°C)^a^	*T*_dec_ (°C)^a^	*λ*_abs_ (nm)^b^	*λ*_em_ (nm)^b^	*I*_P_ (eV)^c^	*E*_g_ (eV)^d^	*E*_ea_ (eV)^e^	*µ*_0_ (cm^2^ V^−1^ s^−1^)^f^
V1476	167	403	265, 355	502	5.34	3.02	2.32	9.0 × 10^−6^
V1481	157	389	260, 335, 365	502	5.30	3.07	2.23	2.6 × 10^−6^

^a^ Glass transition (*T*_g_) and decomposition (*T*_dec_) temperatures determined through DSC and TGA, respectively (10℃ min^−1^, N_2_ atmosphere).

^b^ Absorption and emission spectra were recorded for THF solutions with a concentration of10^−4^ M.

^c^ Ionization energies of the films were measured using photoemission of electrons in air (PESA) method.

^d^ The optical bandgap (*E*_g_) was estimated from the intersection of absorption and emission spectra of solid films.

^e^ Electron affinity (*E*_ea_) was calculated as the difference IP – *E*_g_.

^f^ Mobility value at zero field strength.

Another essential characteristic for an effective charge-transporting material is its charge carrier mobility, determining the speed at which electrons or holes move in the device. Normally, hole-mobility values at zero field are 10^−4^ cm^2^ V s^−1^ and higher values are desired for SCs. Xerographic time of flight (XTOF) measurements were employed to measure the charge mobility of the newly developed HTM layers. Experimental data illustrating the dependence of hole-drift mobility on electric field strength are depicted in figure 6*b*. The relationship between hole drift mobility and electric field strength is characterized by a Bässler-type dependence, which is typical for organic HTMs in most cases [40]. The zero-field hole drift mobility of V1476, almost reaching 1 × 10^−5^ cm^2^ V s^−1^, is higher than the hole drift mobility of *meta-*substituted HTM V1481 which is 2 × 10^−6^ cm^2^ V s^−1^. Meanwhile, the mobility values at strong electric fields are approximately 10^−4^ and 10^−5^ cm^2^ V^−1^ s^−1^ for V1476 and V1481, respectively. Both materials are characterized by Gaussian charge transport: the transit time *t_t_* was determined by the kink on the curve of the d*U*/d*t* transient in linear scale (insets in figure 6*c*,*d*). This indicates that the molecules pack closely ensuring efficient charge transfer in the layers of these materials. In the V1481 material, at weaker electric fields, the signal kinetics (figure 6*d*) show slight hole trapping, which may be related to a less ordered packing of the molecules. This is also in accordance with the lower *T*_g_ of V1481 compared with that of V1476.

Table 2 summarizes the thermal, optical and photoelectrical properties of the spirobisindane-based HTMs.

## 4. Conclusions

In this work, two novel spirobisindane-based enamines were designed and synthesized from commercially available starting materials. Following a comprehensive assessment of their thermal, optical and photophysical properties, and a comparative analysis with those of the HTMs utilized in SCs reported in the scientific literature, it is evident that compounds V1476 and V1481 emerge as promising candidates for applications in organic or hybrid electronics. The synthesized materials exhibit noteworthy thermal and electrochemical stability, possess suitable energy levels and demonstrate sufficiently high drift carrier mobility, reaching 10^−4^ cm^2^ V^−1^ s^−1^ (V1476) at strong electric fields. These characteristics position them favourably as HTMs for use in perovskite SCs and antimony selenide SCs. The experimental findings were complemented by the DFT Cam-B3LYP method. It has been determined that, in the three most probable molecular conformations, the central core fragment does not participate in charge transfer excitations, and the molecular charge redistribution occurs solely between substituents.

## Data Availability

Supporting information is available online at Dryad [41]. Electronic supplementary material is available online [42].
